# Incidence and predictors of loss to follow-up among adult patients receiving antiretroviral therapy in Central Ethiopia: a multi-center retrospective cohort study

**DOI:** 10.3389/fpubh.2024.1374515

**Published:** 2024-03-13

**Authors:** Asfaw Anulo, Addisu Girma, Gezahegn Tesfaye, Fekede Asefa, Abera Cheru, Arega Abebe Lonsako

**Affiliations:** ^1^Dr Bogalech Gebre Memorial General Hospital, Durame, Ethiopia; ^2^College of Health and Medical Science, Haramaya University, Harar, Ethiopia; ^3^Department of Pediatrics, The University of Tennessee Health Science Center (UTHSC) – Oak Ridge National Laboratory (ORNL) Center for Biomedical Informatics, Memphis, TN, United States; ^4^School of Environmental Health Science, College of Health and Medical Science, Haramaya University, Harar, Ethiopia; ^5^College of Medicine and Health Science, Arba Minch University, Arba Minch, Ethiopia

**Keywords:** adults, incidence, antiretroviral therapy, LTFU, predictors

## Abstract

**Background:**

Globally, loss to follow-up (LTFU) remains a significant public health concern despite the rapid expansion of antiretroviral medication programs. It is a significant cause of treatment failure and threatens the enhancement of HIV treatment outcomes among patients on antiretroviral therapy (ART). However, there is a paucity of evidence on its incidence and predictors in Ethiopia. Thus, this study aimed to examine the incidence and predictors of LTFU among adult HIV patients receiving ART at hospitals in Central Ethiopia.

**Methods:**

A multi-centered facility-based retrospective cohort study was conducted among 432 randomly selected adult patients who received antiretroviral therapy. Data were entered into EpiData version 3.1 and exported to Stata version 14 for analysis. The Kaplan–Meier failure function was employed to determine the overall failure estimates, and the log-rank test was used to compare the probability of failure among the different categories of variables. The Cox proportional hazard model was used to identify independent predictors of LTFU.

**Results:**

Overall, 172 (39.8%) study participants were lost to follow-up over the 10-year follow-up period with an incidence rate of 8.12 (95% CI: 7.11, 9.09) per 1,000 person-months. Undisclosed HIV status (AHR: 1.96, 95% CI: 1.14, 3.36), not able to work (AHR: 1.84, 95% CI: 1.13, 2.22), opportunistic infections (AHR: 3.13, 95% CI: 2.17, 4.52), CD4 < 200 cell/mL (AHR: 1.95, 95% CI: 1.18, 3.21), not receiving isoniazid preventive therapy (IPT) (AHR: 2.57, 95% CI: 1.62, 4.06), not participating in clubs (AHR: 1.68, 95% CI: 1.10, 2.22), side effects of drugs (AHR: 1.44, 95% CI: 1.02, 2.04), and high viral load (AHR: 3.15, 95% CI: 1.81, 5.47) were identified as significant predictors of loss to follow-up.

**Conclusion:**

In this study, the incidence of LTFU was high. The focus should be on creating awareness and prevention programs that aim to reduce loss to follow-up by continuing counseling, especially on the negative effects of loss to follow-up and the benefits of ART care.

## Introduction

With advancements and expansion in antiretroviral therapy and the growing access to and use of human immunodeficiency virus (HIV) medical services over almost two decades, a notable positive change has been observed worldwide in people living with HIV/AIDS (PLWH) (1). HIV infection with successful antiretroviral treatment can now be considered a manageable chronic disease in most settings (2).

One of the main obstacles to successful HIV patient care and treatment is the loss to follow-up (LTFU) from antiretroviral therapy (ART) care (3). LTFU is defined as the absence of an ART refill or follow-up for 3 months or longer since their last follow-up schedule (4).

Studies have shown that the incidence of LTFU varies worldwide. A study conducted in Nigeria reported an overall incidence of 55.6 per 100 person-years (5). A similar study conducted in southern Uganda reported an overall incidence rate of 26.7 per 100 person-years (6). However, Ethiopia differs at regional and facility levels. Previous studies conducted in the Amhara region (7) and Arba Minch General Hospital (8) showed that the overall incidence of LTFU was 13.45 per 100 person-years and 5.3 per 100 person-years, respectively.

Different studies have identified factors that predict LTFU from ART clinics, including male sex, age group 15–24, poor/fair adherence, WHO clinical stage III/IV, low CD4, not receiving isoniazid preventive therapy (IPT), and cotrimoxazole preventive therapy (CPT). Similarly, studies have shown that alcohol and substance abuse, TB and other opportunistic infections, stigma, rural residence, demotivation of the service, increased transportation cost, long distance, and waiting time were other contributors to LTFU (9–14).

The loss to follow-up can have negative consequences for patients with HIV. If patients’ LTFU interrupt ART, their immunological and clinical conditions can deteriorate and lead to treatment failure and eventually to increased AIDS mortality. Additionally, it has detrimental effects such as medication resistance, drug toxicity, and potential transmission of the virus. Furthermore, it is a significant obstacle for program implementers as it suggests a poor utilization of limited resources, such as treatment (14, 15). However, there is a paucity of evidence on its incidence and predictors in Ethiopia in general, and there is no specific evidence on it in the study area. Thus, this study aimed to examine the incidence and predictors of LTFU among adult human immunodeficiency virus (HIV) patients receiving ART in Central Ethiopia.

## Materials and methods

### Study design, setting, and period

We conducted a multi-centered facility-based retrospective cohort study in hospitals of Kembata zone and Tembaro Special District in Central Ethiopia. The Kembata zone is located 340 km southwest of the capital city of Ethiopia, Addis Ababa, and 130 km from Hawassa. According to the 2019 Zonal Report, the estimated total population was approximately 941,313, of which 498,896 were men and 442,417 were women. The Kembata zone has four governmental hospitals (Dr Bogalech Gebre Memorial General Hospital, Doyogana Primary Hospital, Shinshicho Primary Hospital, and Angecha Primary Hospital), while Tembaro Special District has one hospital (Mudula Primary Hospital). All of these hospitals provide ART services, except Angecha Primary Hospital. According to the Kembata zone Health Management Information System (HMIS) 2020 Report, these hospitals provided antiretroviral (ARV) services to 1,301 patients, and out of which 1,194 were adults. Patients’ medical records from January 2010 to December 2020 were extracted from 1 June 2022 to 30 June 2022.

### Populations

All HIV-positive adult patients who had at least one treatment follow-up from 1 January 2010 to 31 December 2020 in the hospitals of Kembata zone and Tembaro Special District were considered as the source population, while all adults with advanced and non-advanced clinical stages at ART enrolment from 1 January 2010 to 31 December 2020 in the hospitals of Kembata zone and Tembaro Special District were considered as the study population.

### Eligibility criteria

All HIV-positive adult patients aged ≥15 years who were enrolled in ART between January 2010 and December 2020 at the selected hospitals were included in this study. However, patients whose registration records did not show the initiation date, whose baseline record data were unavailable, who had undefined outcomes, and who had been transferred in with incomplete baseline data were excluded from this study.

### Sample size determination

The sample size was determined using a double population proportion formula considering the advanced stage with a proportion of 0.29 vs. non-advanced stage with a proportion of 0.16 as an independent predictor (13). We set α = 5, power = 80%, and a withdrawal rate of 10% for incomplete data, with an allocation ratio of 1:1 and a standard deviation of 0.5. The estimated sample size for the survivor function was 432. Of these, 216 had advanced WHO clinical stages, and 216 had non-advanced WHO disease stages.

### Sampling technique

The participants were selected from all public hospitals that provided antiretroviral therapy services in the Kembata zone and Tembaro Special District. A list of potential participants was obtained from the adult ART register of each hospital. The total number of ART patient medical records and charts in these hospitals from January 2010 to December 2020 was 894, of which 467 were recruited at non-advanced disease stages. The number of patients who were recruited from each hospital were proportionally allocated based on the patient load to obtain the final sample size. In addition, the sample was collected using a simple random sampling method by taking the list of all ART patients’ medical records and charts and using their unique ART number in each hospital as a sampling frame.

### Operational definitions

Event: LTFU is defined as the absence of an ART refill or follow-up for 3 months or longer since the last follow-up schedule and not reported or recorded as dead or transferred on the patient’s logbook or medical cards (4).

Censored: Patients who did not LTFU until the study ended were recorded as dead and were transferred out (16).

Survival time refers to the total time in months from the initiation of antiretroviral therapy to the occurrence of an event or censored (16).

Advanced disease stage is defined as adults and adolescents with CD4 cell counts of less than 200 cells/mm^3^ or WHO stage 3 or 4 events (14).

Good adherence is defined as patients who took ≥95% of doses or missed ≤2 doses out of 30 doses per month and less than or equal to 3 doses out of 60 doses per month.

Poor adherence is defined as patients taking less than 85% of doses or missing more than five doses out of 30 doses per month and more than nine doses out of 60 doses per month (14).

Appointment spacing model (ASM) or differentiated service delivery (DSD) is a 6-month multi-month dispensing (6-MMD) model in which stable ART patients visit health facilities twice a year for clinical evaluation and laboratory testing when needed; during this visit, they receive 6 months’ worth of ART (14).

### Data collection and quality control

Data were extracted using an English checklist based on nationally standardized ART intake, follow-up, and ART registers. Four health professionals (two BSc nurses and two public health officers) experienced in ART were recruited as data collectors. The patients received 2 days of training on the objectives of the study, the contents of the checklist, and how to review patients’ documents. Two supervisors (one public health expert and one data clerk) participated during the data collection period to supervise the overall data collection process. The entire data collection process was closely monitored daily by supervisors and the principal investigators.

### Data processing and analysis

After data collection, the investigator coded, checked, and cleaned the data. The data were entered into EpiData version 3.1 and then exported to Stata version 14.0 for data processing and analysis. The outcome variables were re-coded as dichotomous outcomes: respondents were lost or not lost. Descriptive statistics such as median, percentage, and frequency were computed and were presented using text, tables, and graphs. A life table was used to estimate the probability of LTFU every 12, 24, 36, 48, 60, 72, 84, 96, or more months. The Kaplan–Meier failure curve was used to estimate the cumulative probability of LTFU after ART initiation. The log-rank test was used to compare significant differences between the groups and was considered statistically significant at *p* < 0.05.

A bivariable Cox regression analysis was performed to determine candidate variables for the multivariable Cox regression model. Variables with a *p* < 0.25 in the bivariable Cox regression model were entered into the multivariable Cox regression model to identify the predictors of incidence of LTFU. The Schoenfeld residuals test (both global and scaled) and graphical (log–log plot of survival) methods were used to check the proportional hazard (PH) assumption, and the overall full model did not violate the proportional hazard assumption. In addition, the most parsimonious model was selected using the log-likelihood ratio, Akaike information criteria (AIC), and Bayesian information criteria (BIC), and the presence of multicollinearity was checked using the variance inflation factor. A *p* < 0.05 was used to declare statistical significance in the multivariable Cox regression model, and an adjusted hazard ratio (AHR) with its 95% confidence interval was computed to show the strength of the association. Moreover, the goodness of fit of the model was assessed using the Cox–Snell residual technique.

## Results

### Sociodemographic characteristics

A total of 432 medical records of adults who received ART between January 2010 and December 2020 were reviewed. The median age of the participants was 32 years, with an interquartile range (IQR) of 28–40 years, and nearly 40% of the participants were in the age group of 25 to 34 years. More than half of the study participants (54.4%) were women, 58.8% of the participants were urban dwellers, and 56.5% of the participants were married. Furthermore, nearly two-thirds of the study participants had attended primary school and above (Table 1).

**Table 1:** Sociodemographic characters of HIV-positive adults receiving ART at hospitals of Kembata zone and Tembaro special district in Central Ethiopia from 1 January 2010 to 31 December 2020 (*n* = 432).

Variables	Categories	Frequency (*n*)	Percentage (%)
Sex	Male	197	45.6
	Female	235	54.4
Age by years	15–24	66	15.3
	25–34	169	39.1
	35–44	136	31.5
	45–54	51	11.8
	55–64	7	1.6
	65 and above	3	0.7
Residence	Urban	254	58.8
	Rural	178	41.2
Religion	Protestant	300	69.4
	Orthodox	91	21.1
	Catholic	26	6.0
	Muslim	15	3.5
Educational level	No formal education	150	34.8
	Primary school	189	43.7
	Secondary school	65	15.0
	College and above	28	6.5
Marital status	Single	107	24.8
	Married	244	56.5
	Divorced	38	8.8
	Widowed	43	9.9
Occupation	Student	63	14.6
	Farmer	136	31.5
	Housewife	148	34.3
	Merchant	51	11.8
	Governmental employer	34	7.8
Distance of facilities	Less than or equal to 20 km	258	59.7
	Greater than 20 km	174	40.3

### Psychosocial and behavioral characteristics

In this study, 46.3% of participants had other HIV-positive family members, while the majority (59.3%) of the participants were living with their parents. Approximately 56.9% of participants disclosed their HIV status. More than three-fourths (80.6%) of the study participants had no history of alcohol use, while 94.0% of participants had no history of smoking cigarettes (Table 2).

**Table 2:** Psychosocial and behavioral characteristics of HIV-positive adults using ART at hospitals of Kembata zone and Tembaro special district in Central Ethiopia from 1 January 2010 to 31 December 2020 (*n* = 432).

Variables	Categories	Frequency (*n*)	Percentage (%)
Another HIV-positive family members	Yes	200	46.3
	No	232	53.7
Living condition	With parent	256	59.3
	Alone	176	40.7
Career	Parent	242	56.0
	Self	190	44.0
Participate in clubs	Yes	159	36.8
	No	240	55.5
Disclosure HIV status	Yes	246	56.9
	No	170	39.4
Disclosed to	Parent	118	27.3
	Friends	17	3.9
	Co-worker	7	1.6
	Husband/wife	114	26.4
Alcohol use	Yes	84	19.4
	No	348	80.6
Smoking	Yes	26	6.0
	No	406	94.0
Adherence	Good	365	84.5
	Poor	67	15.5

### Clinical and treatment-related characteristics

The majority (62.0%) of the participants had a normal body mass index (BMI), and 87.5% of the participants had a working functional status. More than half (55.9%) of the participants had CD4 counts 350 cell/mm^3^ and above. Most (84.3%) of the participants had taken the isoniazid therapy. More than half (53.2) of the participants had at least one opportunistic infection, and 23.8, 19.8, and 10.9% of the participants developed tuberculosis, pneumonia, and zoster, respectively (Table 3).

**Table 3:** Clinical and treatment-related characteristics of HIV-positive adults using ART at hospitals of Kembata zone and Tembaro special district in Central Ethiopia from 1 January 2010 to 31 December 2020 (*n* = 432).

Variables	Categories	Number (*n*)	Percent (%)
Body Mass Index	Underweight	93	21.5
	Normal	268	62.0
	Over/obese	71	16.4
Functional status	Working	378	87.5
	Ambulatory	50	11.6
	Bedridden	4	0.9
CD4 count in mm^3^	≤200 cells	91	21.1
	200–350 cells	104	24.1
	≥350 cells	237	55.9
Isoniazid therapy	Yes	364	84.3
	No	68	15.7
CPT	Yes	328	75.9
	No	104	24.1
Opportunistic infection (OI)	Yes	230	53.2
	No	202	46.8
OI Types	Zoster	47	10.9
	Pneumonia	85	19.8
	Tuberculosis	103	23.8
Time started	Same day	101	23.4
	Within 2 weeks	130	30.1
	Within 4 weeks	77	17.8
	More than 4 weeks	124	28.7
Types of regimen	1C	65	15.0
	1D	32	7.4
	1E	172	39.8
	1F	35	8.1
	1 J	120	27.8
	Other	8	1.8
Number of regimen	One	357	82.6
	Two	75	17.4
Drug side effect	Yes	103	23.8
	No	329	76.2
Follow-up type	Routine	266	61.6
	ASM	166	38.4
Viral load	<1,000	245	56.7
	≥1,000	34	7.9
	Not done	153	35.4
LTFU	Yes	172	39.8
	No	260	60.2

1C = AZT + 3tc + nvp, 1D = AZT + 3tc + efv, 1E = tdf + 3tc + efv, 1F = tdf + 3tc + nvp, 1 J = tdf + 3tc + dtg. 1C, 1E, and 1J were single or one fixed tablet and 1D, 1F, 2F, 2G were double or two fixed tablets.

### Incidence of LTFU among adult HIV patients on ART

**Figure 1:**
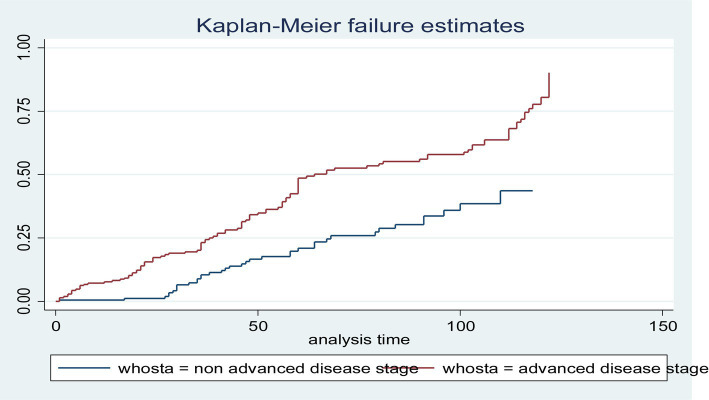
The Kaplan–Meier failure function of loss to follow-up among HIV-positive adults using ART over their WHO stage categories from 1 January 2010 to 31 December 2020 (*n* = 432).

In this study, all (432) participants contributed a total of 21,175 person-months, of which 11,227 person-months contributed to patients with advanced disease. In this study, the incidence rate of LTFU was 8.12 (95% CI: 7.11–9.09) per 1,000 person-months. The incidence rate of LTFU with advanced disease stage was 10.15 (95% CI: 9.21–11.19) per 1,000 person-months. Similarly, the incidence rate of LTFU with non-advanced disease stage was 5.83 (95% CI: 4.72–6.91) per 1,000 person-months. The total proportion of the patients with LTFU was 172 (39.8, 95% CI: 35.3–44.5); of these, 114 (52.8, 95% CI: 37.2–56.5) and 58 (26.85, 95% CI: 32.3–40.2) of them had advanced and non-advanced disease stages, respectively. The overall probability of LTFU in the non-advanced disease stage cohort was significantly different from that in the advanced disease stage cohort. This means that the risk of LTFU was higher in the advanced disease stage cohort than in the non-advanced disease stage cohort (log-rank = 0.003) (Figure 1).

**Figure 2:**
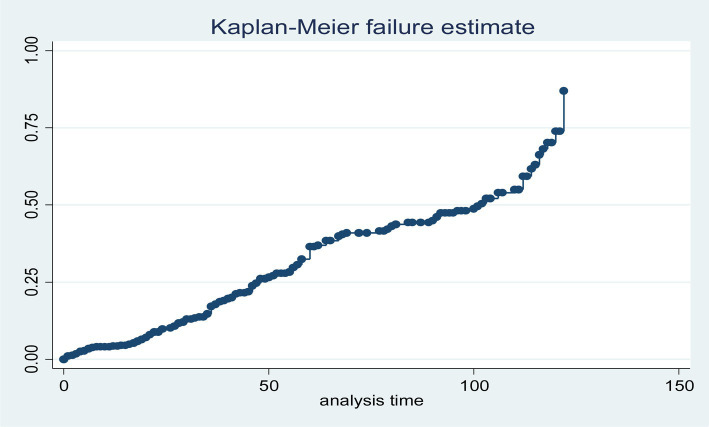
Cumulative failure function of loss to follow-up among HIV-positive adults using ART at hospitals of Kembata zone and Tembaro Special District in Sentral Ethiopia, from 1 January 2010 to 31 December 2020 (*n* = 432).

The overall graph of the Kaplan–Meier failure function shows that the likelihood of LTFU increased over a follow-up period (Figure 2).

The median time for patients to be LTFU receiving ART treatments was 14.25 months. However, the findings of this study revealed that there were differences in the median time of LTFU between advanced and non-advanced disease stage patients, with 16.78 and 12.67 months, respectively.

### Predictors of LTFU among adult HIV patients receiving ART

To determine the predictors of LTFU, variables from bivariate Cox regression analysis (*p* < 0.25) were received as candidate variables for multivariate Cox regression analysis. Having other HIV-positive family members, living alone, being a self-caregiver, participating in clubs, disclosing HIV status, drug adherence status, functional status, BMI, viral load >1,000 copies/ml, follow-up type, OI, CD4 count <200 cells/mm^3^, not receiving IPT, and drug-related side effects were significantly associated with LTFU at *p* < 0.25.

In multivariable Cox regression analysis, HIV status disclosure, participation in clubs, functional status, viral load >1,000 copies/ml, presence of OI, CD4 count <200 cell/mm^3^, presence of drug-related side effects, and not receiving IPT were significant predictors of an increased risk of LTFU. The risk of LTFU among patients who did not disclose their HIV status was 1.94 (95% CI: 1.12–3.36) times higher than that among those who disclosed their HIV status. Patients with non-working functional status were 1.84 (95% CI: 1.13–2.22) times more likely to have LTFU than those with working functional status. Furthermore, patients who did not receive IPT were more likely to be LTFU than those who received IPT (AHR = 2.53, 95% CI: 1.62–4.06).

In addition, the risk of LTFU was higher in patients with CD4 counts ≤200 cells/ mm^3^ compared to those with CD4 counts ≥350 cells/mm^3^ (AHR = 1.95, 95% CI: 1.19–3.22). Similarly, patients who developed opportunistic infection were 3.13 (95%CI: 2.08–4.30) times at risk of loss from care than those who did not develop any opportunistic infection. Furthermore, the risk of LTFU was increased by 44% among patients who developed drug-related side effects as compared with patients who did not develop drug-related side effects (AHR = 1.44, 95% CI: 1.02–2.04). Conversely, patients who did not participate in clubs were 1.69 (95% CI, 1.09–2.62) times more at risk of loss from care than patients participating in clubs. Furthermore, patients with a high viral load had a 3.145 times increased risk of LTFU compared to patients with a viral load of less than 1,000 copies/ml (AHR = 3.145, 95% CI: 1.808–5.469) (Table 4).

**Table 4:** Predictors of LTFU among HIV-positive adults using ART at hospitals of Kembata zone and Tembaro special district in Central Ethiopia, 2022 (*n* = 432).

Variables	Categories	LTFU		CHR (95% CI)	AHR (95% CI)
		Yes	No		
Other HIV-Positive Family Members	Yes	58	142	Ref	Ref
	No	114	118	1.76 (1.28, 2.42)	1.39 (0.86, 2.22)
Disclose HIV Status	Yes	72	89	Ref	Ref
	No	100	171	2.24 (1.65, 2.03)	1.96 (1.14, 3.36)*
Living Condition	With parents	37	122	Ref	Ref
	Alone	135	138	1.87 (1.38, 2.53)	0.85 (0.41, 1.72)
Caregiver	Self	72	171	1.93 (1.43, 2.61)	0.81 (0.38, 1.78)
	Parents	100	89	Ref	Ref
Participation In Clubs	Yes	76	80	Ref	Ref
	No	96	180	1.87 (1.29, 2.71)	1.69 (1.09, 2.62)*
Adherence to HAART	Good	75	181	Ref	Ref
	Poor	97	79	3.15 (2.26, 4.38)	1.29 (0.87, 1.99)
Functional Status	Working	41	247	Ref	Ref
	Not able to work	131	13	2.27 (1.73, 2.99)	1.84 (1.13, 2.22)*
CD4 Count	<200 cell/ mm^3^	29	62	2.03 (1.38, 2.98)	1.95 (1.18, 3.21)*
	200–350 cell/ml	60	44	5.54 (3.28, 9.36)	0.74 (0.50, 1.08)
	>350 cell/ml	171	66	Ref	Ref
Body Mass Index in Kg/m^2^	18.5–24.4	102	166	Ref	Ref
	<18.4	57	36	3.64 (2.99, 6.68)	1.06 (0.52, 2.12)
	>24.5	13	58	2.74 (1.43, 5.25)	2.04 (0.95, 4.36)
Isoniazid Preventive Therapy	Yes	48	20	Ref	Ref
	No	124	240	6.01 (4.24, 8.52)	2.57 (1.62, 4.06)*
Opportunistic Infection	Yes	122	108	3.07 (2.20, 4.29)	3.13 (2.17, 4.52)*
	No	50	152	Ref	Ref
Drug Side Effect	Yes	57	46	1.72 (1.25, 2.37)	1.44 (1.02, 2.04)*
	No	115	214	Ref	Ref
Types of Follow-up	Routine	120	146	Ref	Ref
	ASM	52	114	1.49 (1.08, 2.07)	1.09(0.076, 1.58)
Viral Load	<1,000 Copies	66	179	Ref	Ref
	≥1,000 Copies	21	13	1.89 (1.16, 3.06)	3.15 (1.81, 5.47)*
	Not done	85	68	1.3 (1.12, 2.13)	1.22 (0.46, 3.21)

^*^p < 0.05 (significance). Ref, reference category; ASM, appointment spacing model; HAART, highly active antiretroviral therapy; LTFU, loss to follow-up.

## Discussion

This study aimed to examine the incidence and predictors of LTFU among adult human immunodeficiency virus (HIV) patients receiving ART at hospitals in Central Ethiopia. The finding revealed that the incidence rate of LTFU was 9.74 per 100 person-years (8.12 per 1,000 person-months) of adult observation in PLHIV receiving ART. Ambulatory functional status, CD4 count less than 200 cells/mm^3^, not participating in clubs, not disclosing HIV status, opportunistic infections (OI), not receiving isoniazid preventive therapy (IPT), developed drug-related side effects, and viral load >1,000 copies/mm^3^ were identified as predictors of LTFU.

The incidence rate in this study was consistent with studies conducted in Gondar, Ethiopia (10.90 per 100-person years) (17), Bahir Dar City, Northwest Ethiopia (9.7 per 100 person-years of observation) (18), Debre birhan, Northeast Ethiopia (8.9 per 100 adult years observation) (19), Gonder, Ethiopia (6.7 per 100 person-years) (20) and Cameroon (9.46 per 100 person-years) (21). On the other hand, this finding was lower compared to a study conducted in other African countries: Uganda (21 per 1,000 person-months) (22), Malawi (26.0 per 100 person-years) (23), Pawi, and Northwest Ethiopia (11.6 per 100-person-years) (24).

In contrast, the findings of this study were higher than those studies conducted in different areas of Ethiopia, Mekele (4.5 per 100 person-years) (25), Debre Markos (3.7 per 100 person-years) (26) and Arba Minch (5.3 per 100 person-years) (8). The difference could be attributed to the difference in total follow-up periods, lifestyles of the communities, socioeconomic differences, variations in sample size, variations in health-seeking behavior, differences in patients’ self-transfer behavior to other health institutions without a standardized form or inappropriate recording, and variations in early death report which could be considered as LTFU.

The current study revealed that adult patients who did not disclose their HIV status were 1.96 times more at risk of LTFU than those who disclosed it. This result is in line with studies conducted in Woldian town (27), Kaffa, and Bench Maji zones; individuals who did not disclose their HIV status had more than twice (AHR = 2.28) the risk of LTFU from ART clinics than those who disclosed their HIV status (28). This variation might be because patients who did not disclose their HIV status could not attend the clinic at the expected level, could not feel safe to pick their medication as scheduled, or could not have enough family support due to the fear of discrimination and stigma, which might lead to LTFU. In addition, there is a fear of rejection and violence by partners, family members, the community, and even in the workplace.

Adult patients whose baseline functional status was ambulatory were 1.84 times more at risk of LTFU than those whose baseline functional status was working. This finding is similar to studies conducted in the Hadiya zone, Ethiopia, where the risk of LTFU in ambulatory patients was higher than that in patients with working functional status (AHR = 2.4) (13). These discrepancies might be because most ambulatory patients were economically dependent. In addition, they required close family and social support, and patients’ self-care activities might be interrupted.

The risk of LTFU was nearly two times higher among patients receiving ART with baseline CD4 counts less than 200 cells/mm^3^ than patients with a baseline CD4 count greater than 350 cells/mm^3^. This finding is in agreement with studies conducted in South Africa (28), Ethiopia (29) and Mizan-Aman General Hospital (30). The reason for this result might be that those with lower CD4 counts have low immunity and therefore develop multiple opportunistic infections and might develop immune reconstitution inflammatory syndrome (IRIS).

HIV-positive patients receiving ART who did not receive isoniazid preventive therapy were 2.6 times more likely to be LTFU than those who received therapy. This result was concordant with a study conducted at Gondar in 2019, which showed that participants with no isoniazid (INH) prophylaxis were 2.47 times more at risk of being LTFU compared to those who took INH (12). Similarly, a study conducted in Mekele on isoniazid preventive therapy (IPT) (AHR = 0.085) reported a protective effect against LTFU (25). This variation might be because those who did not take IPT were at risk of developing active TB, drug toxicity, and drug–drug interactions.

Adult HIV-positive patients with at least one opportunistic infection were three times more likely to be LTFU than those without any opportunistic infection. This finding is more consistent with studies conducted in sub-Saharan Africa (16), Cameron (31) and Gondar Hospital (12) which shows that those who had opportunistic infection were 2.5, 4.75, and 1.74 times more likely to be LTFU, respectively, compared to those who had no opportunistic infection. This discrepancy might be due to illness severity and increased drug-related pill burden as well as drug–drug interactions, toxicity, and adherence of patients to ARV.

Adult patients receiving ART with a high viral load of ≥1,000 copies/mL were 3.15 (95% CI: 1.808–5.469) times at risk of LTFU compared to those with a low viral load of <1,000 copies/mL. Possible explanations for treatment failure include the development of multiple opportunistic infections and the pill burden.

Similarly, adults receiving ART who developed any drug-related side effects were 1.44 (95% CI: 1.02–2.04) times more likely to be LTFU compared to those who had free drug side effects. A possible explanation might be the severity of the illness, fear of stigma, and discrimination. Furthermore, adults who did not participate in any clubs/associations were 1.7 (95% CI: 1.09–2.62) times more at risk of LTFU compared to those who participated in clubs. This discrepancy might also be due to the fear of stigma and discrimination. Therefore, for these three variables, high viral loads, drug-related side effects, and clubs/associations did not show similar results and were not found in the reviewing literature. However, this finding requires further investigation.

This study has some limitations: the study was conducted on secondary data and might have some secondary data limitations, and retrospective analysis can limit more predictive variables of LTFU on the records than on the patient’s side.

## Conclusion

In this study, the incidence of LTFU was high. Patients who did not disclose HIV status, ambulatory functional status, CD4 count less than 200 cells/mm^3^, not participating in clubs, having opportunistic infections, isoniazid preventive therapy, having a viral load greater than 1,000 copies/mL, and those who developed drug-related side effects were significant predictors of LTFU.

## Recommendations

Integration should be considered with multiple sectors and civil society, including partners and organizations, to create awareness and prevention programs that target LTFU. They should provide continuous counseling and health education targeting the negative effects of LTFU and the benefits of ART care. Hospitals should strengthen ART monitoring and follow-up systems to decrease the LTFU and loss tracking methods. In addition, effective patient monitoring systems should be developed, particularly for those with high viral loads, to prevent adverse drug effects.

Future studies should be conducted using prospective and qualitative designs to predict factors of LTFU among PLWH and the outcome of LTFU.

## Data availability statement

The original contributions presented in the study are included in the article/supplementary material, further inquiries can be directed to the corresponding authors.

## Ethics statement

The studies involving humans were approved by Haramaya University College of Health and Medical Sciences Institutional Health Research ethics review committee. The studies were conducted in accordance with the local legislation and institutional requirements. Written informed consent for participation was not required from the participants or the participants’ legal guardians/next of kin in accordance with the national legislation and institutional requirements.

## Author contributions

AA: Conceptualization, Data curation, Formal analysis, Funding acquisition, Investigation, Methodology, Project administration, Resources, Software, Supervision, Validation, Visualization, Writing – original draft, Writing – review & editing. AG: Conceptualization, Data curation, Formal analysis, Funding acquisition, Investigation, Methodology, Project administration, Resources, Software, Supervision, Validation, Visualization, Writing – original draft, Writing – review & editing. GT: Conceptualization, Data curation, Formal analysis, Funding acquisition, Investigation, Methodology, Project administration, Resources, Software, Supervision, Validation, Visualization, Writing – original draft, Writing – review & editing. FA: Project administration, Resources, Software, Supervision, Validation, Visualization, Writing – original draft, Writing – review & editing, Conceptualization, Data curation, Formal analysis, Funding acquisition, Investigation, Methodology. AC: Conceptualization, Data curation, Formal analysis, Funding acquisition, Investigation, Methodology, Project administration, Resources, Software, Supervision, Validation, Visualization, Writing – original draft, Writing – review & editing. AL: Conceptualization, Data curation, Formal analysis, Funding acquisition, Investigation, Methodology, Project administration, Resources, Software, Supervision, Validation, Visualization, Writing – original draft, Writing – review & editing.
